# Contrasting allocation patterns in wheat and weeds: allometric belowground and reproductive investment versus optimal partitioning adaptations

**DOI:** 10.3389/fpls.2025.1542205

**Published:** 2025-04-24

**Authors:** Jiazhen Xi, Shengtao Shi, Yizhong Rong, Jie Liu, Li Zhang

**Affiliations:** ^1^ Co-Innovation Center for Sustainable Forestry in Southern China, Bamboo Research Institute, College of Ecology and the Environment, Nanjing Forestry University, Nanjing, China; ^2^ School of Resources and Environment, Anhui Agricultural University, Hefei, China

**Keywords:** allometric partitioning theory, biomass allocation, plant life history, reproductive strategy, size dependence

## Abstract

**Introduction:**

Modeling differences in biomass allocation between wheat and weeds—specifically to shoots (aboveground biomass), roots (belowground biomass), and seed mass (reproductive biomass)—enhances our understanding of sustainable weeds management. However, few studies have examined how fertilization and planting density influence biomass accumulation and allocation at both vegetative and reproductive stages within a wheat-weed community.

**Methods:**

To address this gap, we conducted a greenhouse experiment growing wheat (*Triticum aestivum* L.), wild oats (*Avena fatua* L.), and barnyard grass (*Echinochloa crusgalli* (L.) P. Beauv.) under varying planting densities (4, 8, 12, and 16 individuals per pot) and fertilization treatments (1.018 g N per pot of urea). After six months of vegetative growth and one additional month at the reproductive stage, we measured aboveground and belowground biomass at both stages, and reproductive biomass during the reproductive stage.

**Results and Discussion:**

We found that the biomass of wheat and weeds increased with fertilization but decreased with higher planting density, with no interactions between these factors. Wheat allocated more biomass to roots than shoots and more to reproductive than vegetative biomass, regardless of fertilization or planting density, following allometric allocation theory. In contrast, weeds distributed biomass similarly between shoots and roots at planting densities of 4 and 12 under fertilization or allocated more biomass to roots than to shoots at these densities. Additionally, some weeds achieved higher yields at both small and large sizes under planting densities of 12 and 16, respectively, suggesting greater phenotypic plasticity. This study provides a comprehensive analysis of biomass allocation differences between wheat and weeds throughout their life cycles, offering insights into plant adaptation strategies and practical applications for optimizing agricultural management.

## 1 Introduction

Wheat plays a critical role in global food security, sustaining over 40% of the world’s population, yet its yield is often reduced due to nutrient competition from weeds (FAO, 2023). Managing weeds diversity has been shown to mitigate crop yield losses (Adeux et al., 2019). Modeling differences in biomass allocation between wheat and weeds can help determine the extent to which human-assisted selection influences specific crop and weeds traits. For both wild and cultivated species, growth and reproduction are fundamental processes in plant life history (Hulshof et al., 2012; Schoen et al., 2019). Plants assimilate carbon and absorb nutrients to build biomass throughout their ontogeny, balancing biomass accumulation between vegetative and reproductive structures (Poorter et al., 2012; Shipley and Meziane, 2002; Yin et al., 2019). However, the patterns of biomass—allocation particularly how wheat and weeds adjust allocation priorities across ontogeny in response to agricultural practices such as fertilization and planting density gradients—remain poorly understood.

Biomass allocation patterns among organs are primarily explained by two major theories: allometric partitioning theory (Enquist and Niklas, 2002; Falster et al., 2015) and optimal partitioning theory (Thornley, 1972). The allometric partitioning theory proposes that the nonlinear, size-dependent distribution of biomass among plant parts remains proportionally consistent across diverse species or community types (Cheng and Niklas, 2007; Cheng et al., 2011; Enquist and Niklas, 2002; Müller et al., 2000; Niklas, 2004; Poorter et al., 2015; Westgeest et al., 2024; Zhou et al., 2014). According to allometric scaling laws, size-related traits in vascular plants follow a power-law function: Y _=_ Y_0_M^b^ or log_10_Y = log_10_Y_0_ + blog_10_M, where Y represents a plant trait, Y_0_ is the trait value when M = 1, M is plant size (typically measured as biomass), and b is the scaling exponent determined by the geometric and hydrodynamic properties of vascular networks, which maximize resource uptake while minimizing transport investment (West et al., 1999). For example, allocating more biomass to vegetation rather than reproduction sustains physiological processes and growth (Brooks, 2003), and enhances competition with neighboring plants (Liu et al., 2023). Conversely, allocating more biomass to reproduction rather than growth increases crop yield and improves the fitness of wild plants (Luo et al., 2020; McCarthy and Enquist, 2007; Pallas et al., 2016; Westgeest et al., 2024; Zheng et al., 2023). The optimal partitioning theory posits that plants allocate more biomass to organs that capture the most limiting resources, thereby enhancing overall plant performance (Bloom et al., 1985).

Numerous studies have examined whether biomass allocation patterns result from optimal partitioning theory or allometric partitioning theory under variable environmental conditions (Fang et al., 2023; Liu et al., 2021; McCarthy and Enquist, 2007; Peng and Yang, 2016). For example, artemisia species adapt to environmental changes through allometric strategies rather than dynamically adjusting organ allocation (Liu et al., 2021). For wheat, significant differences among varieties have been observed in the allometric exponent (the slope of the log-log relationship) of seed versus vegetative biomass. Some varieties, such as DM31 and HST, produce higher yields when individual plants are small, while varieties like MO1 achieve greater yields as individuals grow larger (Qin et al., 2013). For weeds, changes in the shoot-root biomass ratio in response to variations in light (Dias-Filho, 1999), water (Acciaresi and Guiamet, 2010), and nutrient gradient (Ringselle et al., 2017) have been reported. For example, in nutrient poor soils, biomass is predominantly allocated to root development rather than aboveground structures to improve nutrient acquisition efficiency. After fertilization, biomass allocation shifts towards aboveground structures as nutrient availability increases for light competition (Ringselle et al., 2017). However, instead of using ratios, which typically vary with size, it has been proposed that the relationship between plant size and reproductive allocation should be analyzed allometrically (Qin et al., 2013; Weiner, 2004).

Fertilization and planting density are critical agronomic factors that exacerbate N limitation and increase or decrease plant development and reproduction accumulation (Fang et al., 1991; Frink et al., 1999; Huangfu et al., 2022; Jia et al., 2020; LeBauer and Treseder, 2008; Postma et al., 2021; Xia and Wan, 2008; Yoda, 1963). Regarding biomass allocation, wheat may allocate more biomass to reproduction regardless of variations in fertilization and planting density. This tendency could be attributed to selective pressures during domestication that favored consistent yields for this staple food crop (Brancourt-Hulmel et al., 2003). Alternatively, wheat may exhibit flexibility in biomass allocation, as significant differences among varieties have been observed (Qin et al., 2013). Weeds are characterized by their ruderal nature, may adjust their allocation patterns in response to environmental changes, displaying flexible survival and reproductive strategies in unpredictable agricultural landscapes (Grime, 1977).

To clarify ontogenetic shifts in biomass allocation in wheat and weeds across fertilization and planting density gradients, we conducted a greenhouse experiment using planting densities of 4, 8, 12 and 16 individuals per pot, with and without urea fertilization. Wheat (*Triticum aestivum*), wild oat (*Avena fatua*), and barnyard grass (*Echinochloa crus-galli*) were selected as target species. We specifically investigated the following three questions: (i) How do fertilization and planting density affect wheat and weeds aboveground and belowground biomass at the vegetative and reproductive stages, as well as their effects on the reproductive biomass (i.e., seed production) and vegetative biomass (i.e., non-reproductive) at the reproductive stage? (ii) How do fertilization and planting density affect allometric scaling relationships among different organs? (iii) Do the effects of fertilization and planting density on allometric scaling relationships among different organs depend on ontogenetic stage and species identity?

## 2 Materials and methods

### 2.1 Study site

This study was conducted in a greenhouse at Hefei High-tech Agricultural Park, Anhui Province, China (31° 55’N, 117° 12’E). The region experiences a typical subtropical monsoon climate, with an average annual temperature of approximately 16.1°C. The mean annual precipitation is about 1149 mm, with the majority occurring in summer.

### 2.2 Study species

Wheat (*Triticum aestivum* L.), wild oat (*Avena fatua* L.) and barnyard grass (*Echinochloa crusgalli* (L.) P. Beanv) were selected as target species. The tested wheat variety was Yannong 19. Wild oat and barnyard grass were two dominant weeds in wheat fields and accounted for 90% of the relative abundance (Zhang et al., 2019). Seeds were collected from the test site and selected for uniform plumpness, free of insect damage and mildew.

### 2.3 Experiment design

The experiment was divided into six groups, with three receiving urea fertilization and the three serving as controls. Each group consisted of 40 pots, incorporating two competing species pairs (i.e., *T. aestivum* and *A. fatua*, *T. aestivum* and *E. crusgalli*), four planting densities (4, 8, 12, and 16 individuals per pot) and five wheat: weeds proportional gradients at each density (0: 1, 0.25: 0.75, 0.5: 0.5, 0.75: 0.25, and 1: 0) (Zhang and van Kleunen, 2019). The planting density in wheat fields ranges from 80-400 individuals/m² (Fischer et al., 2019). The selected planting densities (4, 8, 12, and 16 individuals per pot) correspond to approximately 110, 220, 330, and 440 individuals/m^2^, respectively, mimicking the structure of natural wheat communities. Among the fertilized groups, two measured biomasses at the vegetative stage, while the third measured biomass at the reproductive stage. The same distribution applied to the non-fertilized groups (**Supplementary Figure S1**).

Seeds of wheat and weeds were soaked in a 5% H_2_O_2_ solution for ten minutes on October 25, 2021, then rinsed with sterile distilled water. The seeds were then placed on wet filter paper to germinate in a plant growth chamber at 25°C. After approximately two weeks, seedlings with similar growth were transplanted into soil pots (24.5 cm in diameter at the top, 18.5 cm at the base, 25 cm in height, 9135 cm^3^ volume). Before transplanting, urea dissolved in water (1.018 g N per pot) was applied to the potting soil (Zhang et al., 2019). Seedlings that died within two weeks after transplanting were replaced, and the plants were watered once every 3-5 days to maintain adequate moisture.

### 2.4 Biomass measurement

Biomass was collected at the individual level during the vegetative stage in May 2022 and the reproductive stage in June 2022 (**Supplementary Table S1**). In May, wheat and weeds seed heads were bagged to prevent seed dispersal. Plant parts were dried at 65°C for 48h and weighed to 0.0001g to determine the dry weight of aboveground, belowground, and reproductive biomass. Vegetative biomass was calculated as the sum of aboveground and belowground biomass.

### 2.5 Statistical analysis

To satisfy the assumption of normality, aboveground, belowground, vegetative and reproductive biomass were log-transformed before analysis. A three-way ANOVA was conducted to assess the effects of ontogenetic stage (vegetative or reproductive), fertilization, and planting density on aboveground and belowground biomass. At each ontogenetic stage, a two-way ANOVA was used to evaluate the effects of fertilization and planting density on aboveground, belowground, vegetative and reproductive biomass. Under each fertilization condition, linear models were used to assess the effects of planting density on biomass. To assess the effects of plant density and fertilization on plants biomass, we constructed linear mixed effects models, with the number of wheat and weeds individuals as fixed effects and pot nested within competing neighbors as a random effect (y ~ Nwheat + Nweeds + (1| Neighbor species/Pot)) under both fertilized and unfertilized conditions.

Standardized major axis (SMA) tests (sma function in SMATR package) were performed according to the allometric equation (Equation 1) after aboveground, belowground, vegetative and reproductive biomass log-transformed (Equation 2) to determine the scaling exponents (slope) under different fertilization and planting density (Huxley, 1972; Wang et al., 2019):


(1) $$\begin{array}{c} Y=\beta X^{\alpha } \end{array}$$

which was usually analyzed:


(2) $$\begin{array}{c} \log Y=\log\beta +\alpha \log X \end{array}$$

where X and Y are the two given biomass, and β is referred to the allometric coefficient. logβ is the intercept and α is the allometric exponent or the slope. α < 1 indicates a faster accumulation in X than Y, and α > 1 indicates a faster accumulation in Y than X. Likelihood ratio tests were used to determine whether the slopes of the SMA estimation varied across different fertilization and planting density (Wang et al., 2022). All statistical analyses were conducted in R 4.2.2 (R Core Team, 2022).

## 3 Results

### 3.1 Changes of aboveground, belowground, vegetative and reproductive biomass with fertilization and planting density

**Figure 1:**
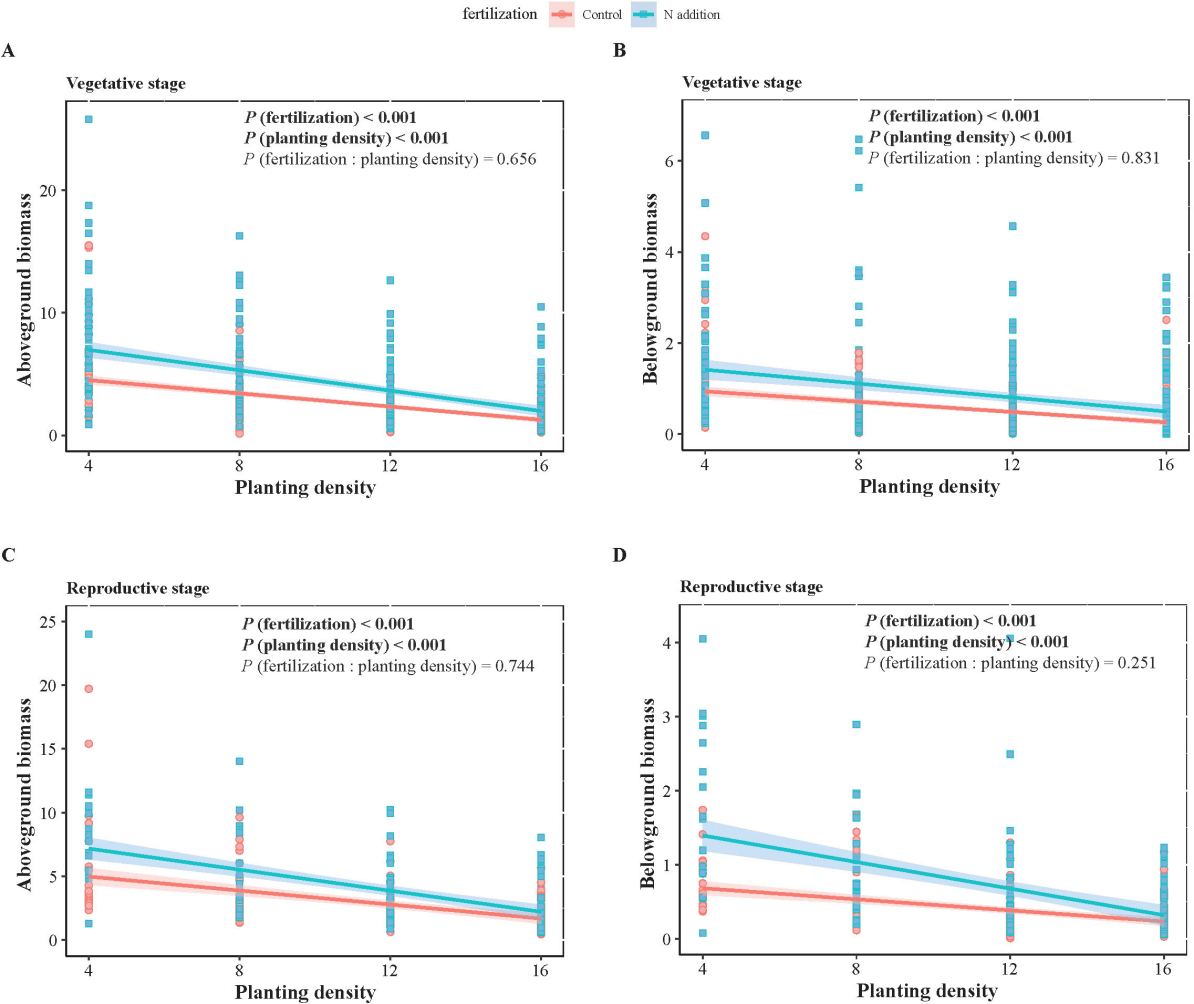
Two-way ANOVA results of fertilization (control and N addition treatments), planting density (4, 8, 12, 16), and their interaction (fertilization: planting density), as well as the effects of planting density under different fertilization conditions on wheat biomass at vegetative **(A, B)** and reproductive **(C, D)** stages. **(A, C)** represent aboveground biomass, while **(B, D)** represent belowground biomass. Solid lines indicate significant linear regressions (*P* < 0.05) with 95% confidence intervals, dashed lines indicate non-significance effect.

**Figure 2:**
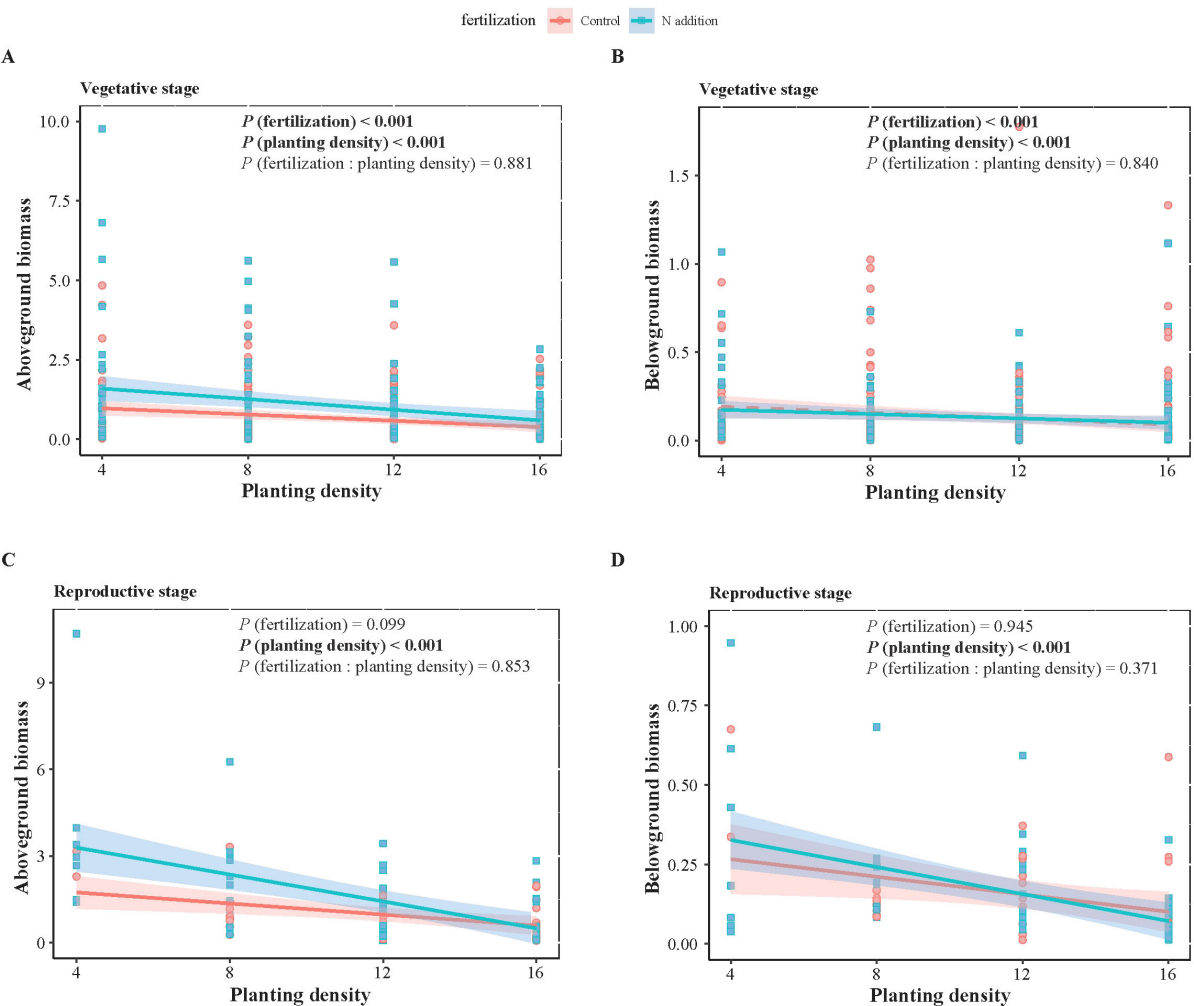
Two-way ANOVA results of fertilization (control and N addition treatments), planting density (4, 8, 12, 16), and their interaction (fertilization: planting density), as well as the effects of planting density under different fertilization conditions on weeds biomass at vegetative **(A, B)** and reproductive **(C, D)** stages. **(A, C)** represent aboveground biomass, while **(B, D)** represent belowground biomass. Solid lines indicate significant linear regressions (*P* < 0.05) with 95% confidence intervals, dashed lines indicate non-significance effect.

**Figure 3:**
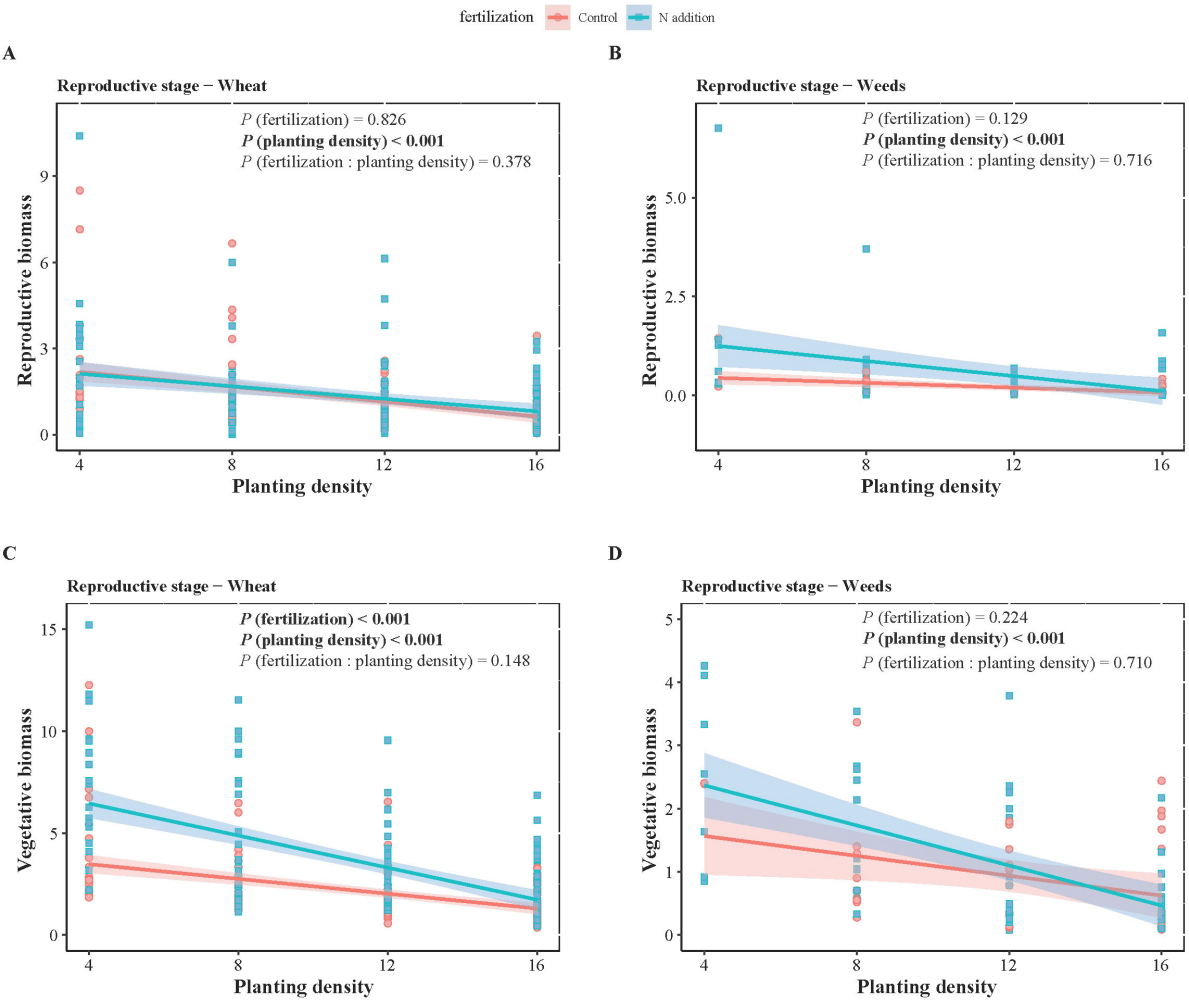
Two-way ANOVA results of fertilization (control and N addition treatments), planting density (4, 8, 12, 16), and their interaction (fertilization: planting density), as well as the effects of planting density under different fertilization conditions on wheat **(A, C)** and weeds **(B, D)** biomass at reproductive stages. **(A, B)** represent reproductive biomass, while **(C, D)** represent vegetative biomass. Solid lines indicate significant linear regressions (*P* < 0.05) with 95% confidence intervals, dashed lines indicate non-significance effect.

For both wheat and weeds, ontogenetic stage (Three-way ANOVA; *F*_1,1068_ = 15.939, *P* < 0.001; *F*_1,1068_ = 4.054, *P* < 0.05; *F*_1,486_ = 34.322, *P* < 0.001; *F*_1,486_ = 15.610, *P* < 0.001), fertilization (*F*_1,1068_ = 118.578, *P* < 0.001; *F*_1,1068_ = 49.189, *P* < 0.001; *F*_1,486_ = 25.813, *P* < 0.001; *F*_1,486_ = 9.558, *P* < 0.01) and planting density (*F*_3,1068_ = 148.600, *P* < 0.001; *F*_3,1068_ = 76.881, *P* < 0.001; *F*_3,486_ = 12.089, *P* < 0.001; *F*_3,486_ = 7.436, *P* < 0.001) significantly affected aboveground and belowground biomass. The interactive effects of these three factors did not significantly influence wheat and weed aboveground and belowground biomass, except for the interaction between ontogenetic stage and fertilization, which significantly affected wheat’s belowground biomass (*F*_1,1068_ = 3.881, *P* < 0.05) (**Tables 1**, **2**). Specifically, fertilization significantly increased wheat aboveground, belowground, and vegetative biomass at both the vegetative and reproductive stages, as well as weeds aboveground and belowground biomass at the vegetative stage. Regardless of fertilization, aboveground, belowground, vegetative, and reproductive biomass both wheat and weeds decreased significantly with increasing planting density (**Figures 1**–**3**; **Supplementary Tables S2**, **S3**, **S4**), except weeds belowground biomass at the vegetative stage in the absence of fertilization (**Figure 2B**). Increasing wheat density significantly reduced both wheat and weeds vegetative and reproductive biomass. However, increasing weeds density only significantly reduced wheat biomass at the vegetative stage and weed reproductive biomass (**Tables 3**, **4**).

**Table 1:** Three-way ANOVA results for the effects of ontogeny (OT), fertilization (FT), planting density (PD) and their interactions on wheat’s M_A_ (aboveground biomass) and M_B_ (belowground biomass).

Factors	M_A_					M_B_				
	*df*	Sum Sq	Mean Sq	*F*	*P*	*df*	Sum Sq	Mean Sq	*F*	*P*
OT	1	0.998	0.998	15.939	**< 0.001**	1	0.563	0.563	4.054	**< 0.05**
FT	1	7.424	7.424	118.578	**< 0.001**	1	6.832	6.832	49.189	**< 0.001**
PD	3	27.912	9.304	148.600	**< 0.001**	3	32.036	10.679	76.881	**< 0.001**
OT: FT	1	0.047	0.047	0.757	0.385	1	0.539	0.539	3.881	**< 0.05**
OT: PD	3	0.142	0.047	0.757	0.518	3	0.317	0.106	0.761	0.516
FT: PD	3	0.099	0.033	0.528	0.663	3	0.428	0.143	1.028	0.379
OT: FT: PD	3	0.075	0.025	0.400	0.753	3	0.159	0.053	0.383	0.766
Residuals	1068	66.870	0.063			1068	148.343	0.139		

Degrees-of-freedom (*df*), sum of squares (Sum Sq), mean square (Mean Sq), *F*-values (*F*) and *P*-values (*P*) are shown. Significant responses are highlighted in bold (*P* < 0.05).

**Table 2:** Three-way ANOVA results for the effects of ontogeny (OT), fertilization (FT), planting density (PD) and their interactions on weeds’ M_A_ (aboveground biomass) and M_B_ (belowground biomass).

Factors	M_A_					M_B_				
	*df*	Sum Sq	Mean Sq	*F*	*P*	*df*	Sum Sq	Mean Sq	*F*	*P*
OT	1	13.417	13.417	34.322	**< 0.001**	1	5.135	5.136	15.610	**< 0.001**
FT	1	10.090	10.091	25.813	**< 0.001**	1	3.145	3.145	9.558	**< 0.01**
PD	3	14.177	4.726	12.089	**< 0.001**	3	7.339	2.446	7.436	**< 0.001**
OT: FT	1	0.787	0.787	2.013	0.157	1	0.832	0.832	2.529	0.112
OT: PD	3	2.220	0.740	1.893	0.130	3	1.494	0.498	1.514	0.210
FT: PD	3	0.200	0.067	0.171	0.916	3	0.074	0.025	0.075	0.973
OT: FT: PD	3	0.221	0.074	0.188	0.904	3	0.709	0.236	0.718	0.973
Residuals	486	189.978	0.391			486	159.889	0.329		

Degrees-of-freedom (*df*), sum of squares (Sum Sq), mean square (Mean Sq), *F*-values (*F*) and *P*-values (*P*) are shown. Significant responses are highlighted in bold (*P* < 0.05).

**Table 3:** Effects of wheat and weeds density on wheat’s aboveground, belowground, vegetative, and reproductive biomass with or without urea fertilization at vegetative and reproductive stages.

Treatment				Individual number of wheats		Individual number of weeds	
			*Intercept*	*Slope*	*P*	*Slope*	*P*
Vegetative stage	Aboveground Biomass	Control	0.950	-0.052	**< 0.001**	-0.022	**< 0.001**
		N addition	1.140	-0.057	**< 0.001**	-0.020	**< 0.01**
	Belowground Biomass	Control	0.187	-0.058	**< 0.001**	-0.015	**< 0.05**
		N addition	0.282	-0.071	**< 0.001**	0.021	**< 0.05**
Reproductive stage	Aboveground Biomass	Control	0.991	-0.055	**< 0.001**	-0.007	0.322
		N addition	1.124	-0.060	**< 0.001**	-0.022	**< 0.01**
	Belowground Biomass	Control	0.105	-0.063	**< 0.001**	-0.008	0.346
		N addition	0.336	-0.069	**< 0.001**	-0.019	0.066
	Vegetative Biomass	Control	0.828	-0.050	**< 0.001**	-0.008	0.233
		N addition	1.099	-0.068	**< 0.001**	-0.023	**< 0.01**
	Reproductive Biomass	Control	0.605	-0.062	**< 0.001**	-0.006	0.369
		N addition	0.388	-0.035	**< 0.05**	-0.022	0.193

Statistically significant responses (P < 0.05) are highlighted in bold.

**Table 4:** Effects of wheat and weeds density on weeds’ aboveground, belowground, vegetative, and reproductive biomass with or without urea fertilization at vegetative and reproductive stages.

Treatment				Individual number of wheats		Individual number of weeds	
			*Intercept*	*Slope*	*P*	*Slope*	*P*
Vegetative stage	Aboveground Biomass	Control	-0.052	-0.094	**< 0.001**	-0.001	0.969
		N addition	0.368	-0.123	**< 0.001**	-0.024	0.101
	Belowground Biomass	Control	-0.813	-0.061	**< 0.05**	0.001	0.970
		N addition	-0.494	-0.059	**< 0.01**	-0.021	0.094
Reproductive stage	Aboveground Biomass	Control	0.538	-0.145	**< 0.05**	-0.053	**< 0.01**
		N addition	0.767	-0.071	**< 0.01**	-0.069	**< 0.001**
	Belowground Biomass	Control	-0.571	-0.217	**< 0.01**	-0.024	0.055
		N addition	-0.288	-0.094	**< 0.01**	-0.034	0.064
	Vegetative Biomass	Control	0.354	-0.153	**< 0.05**	-0.041	**< 0.05**
		N addition	0.670	-0.072	**< 0.01**	-0.057	**< 0.01**
	Reproductive Biomass	Control	-0.021	-0.109	0.059	-0.065	**< 0.05**
		N addition	0.139	-0.084	**< 0.05**	-0.076	**< 0.01**

Statistically significant responses (P < 0.05) are highlighted in bold.

### 3.2 Wheat allometric relationships between aboveground and belowground biomass

At the vegetative stage, the scaling exponents (α) for wheat aboveground biomass versus belowground biomass were 1.375, 1.295, 1.443, 1.290 at planting densities of 4, 8, 12, 16, respectively, without fertilization. With fertilization, the α values were 1.175, 1.704, 1.684, 1.750 for the same respective densities. At the reproductive stage, the α values were 1.208, 1.879, and 1.338 at planting densities of 8, 12, and 16, respectively, without fertilization, and 1.410, 1.533, and 1.378 under fertilization. Additionally, at a planting density of 4, the α value was 0.711 under non-fertilized conditions at the reproductive stage (**Figure 1**, **Table 5**).

**Table 5:** Scaling exponents (α), their 95% confidence intervals (95% CI), and goodness of fit (R^2^) for wheat, based on standardized major axis (SMA) regression of log-transformed aboveground biomass (M_A_)and belowground biomass (M_B_) under different fertilization condition (control and fertilization) and planting densities (4, 8, 12 and 16) at vegetative and reproductive stages. *N* represents individual number. *P* values indicate the significance of SMA between the two organs.

Species	Treatment	Planting density	M_A_ vs M_B_ at vegetative stage					M_A_ vs M_B_ at reproductive stage				
			*N*	α	95% CI	*R^2^*	*P*	*N*	α	95% CI	*R^2^*	*P*
Wheat	Control	4	39	1.375	1.103, 1.715	0.555	**< 0.001**	19	$0.711_{\text{B}}^{\text{c}}$	0.520, 0.972	0.614	**< 0.001**
		8	77	1.295_B_	1.054, 1.592	0.186	**< 0.001**	33	1.208^b^	0.916, 1.594	0.414	**< 0.001**
		12	117	1.443	1.232, 1.690	0.260	**< 0.001**	56	1.879^a^	1.465, 2.409	0.152	**< 0.01**
		16	149	1.290_B_	1.116, 1.491	0.206	**< 0.001**	72	1.338^ab^	1.131, 1.583	0.496	**< 0.001**
	Fertilization	4	40	1.175^b^	0.883, 1.563	0.223	**< 0.01**	18	$1.569_{\text{A}}^{\text{ab}}$	0.950, 2.591	0.026	0.524
		8	75	$1.704_{\text{A}}^{\text{a}}$	1.425, 2.038	0.405	**< 0.001**	32	1.410^ab^	1.093, 1.818	0.524	**< 0.001**
		12	105	1.684^a^	1.411, 2.010	0.170	**< 0.001**	53	1.533^a^	1.181, 1.991	0.116	**< 0.05**
		16	139	$1.750_{\text{A}}^{\text{a}}$	1.493, 2.052	0.107	**< 0.001**	60	1.378^b^	1.135, 1.674	0.448	**< 0.001**

Different superscript characters indicate significant differences among planting density under a given fertilization condition, and different subscript characters indicate significant differences among fertilization condition under a given planting density. Significant responses are highlighted in bold (*P* < 0.05).

### 3.3 Weeds allometric relationships between aboveground and belowground biomass

At the vegetative stage, weeds exhibited similar biomass allocation between aboveground and belowground biomass under certain conditions. The scaling exponents (α) were 1.099, 0.901, 1.004, and 0.918 at planting densities of 4, 8, 12, and 16, respectively, under non-fertilized conditions. With fertilization, the α values were 0.918 and 1.082 at planting densities of 4 and 12, respectively. At the reproductive stage, the α values were 0.837 at a planting density of 16 without fertilization and 0.880 at a density of 12 with fertilization. For planting density of 8 and 16 with fertilization at the vegetative stage, and 8 without fertilization at the reproductive stage, α were 0.700, 0.747, and 0.491, respectively. At a planting density of 12 without fertilization at the reproductive stage, α was 1.368. At the reproductive stage, for planting densities of 4, 8 and 16 with fertilization, α values did not show significance (*P* = 0.325; *P* = 0.432; *P* = 0.067) (**Figure 2**, **Table 6**).

**Table 6:** Scaling exponents (α), their 95% confidence intervals (95% CI), and goodness of fit (R^2^) for weeds based on standardized major axis (SMA) regression of log-transformed M_A_ and M_B_ under different fertilization conditions (control and fertilization) and planting densities (4, 8, 12 and 16) at vegetative and reproductive stages. *N* represents individual number. *P* values indicate the significance of SMA between the two organs.

Species	Treatment	Planting density	M_A_ vs M_B_ at vegetative stage					M_A_ vs M_B_ at reproductive stage				
			*N*	α	95% CI	*R^2^*	*P*	*N*	α	95% CI	*R^2^*	*P*
Weeds	Control	4	25	1.099	0.902, 1.338	0.788	**< 0.001**	2	*	*	*	*
		8	55	0.901	0.781, 1.039	0.731	**< 0.001**	10	0.491^b^	0.344, 0.700	0.802	**< 0.001**
		12	65	1.004	0.874, 1.155	0.690	**< 0.001**	11	$1.368_{\text{A}}^{\text{a}}$	1.007, 1.859	0.830	**< 0.001**
		16	85	0.918	0.805, 1.047	0.634	**< 0.001**	17	0.837^a^	0.554, 1.265	0.406	**< 0.01**
	Fertilization	4	25	0.918^ab^	0.714, 1.180	0.654	**< 0.001**	7	1.826	0.739, 4.511	0.192	0.325
		8	48	0.700^b^	0.550, 0.891	0.327	**< 0.001**	11	0.658	0.335, 1.292	0.070	0.432
		12	39	1.082^a^	0.840, 1.394	0.410	**< 0.001**	17	0.880_B_	0.741, 1.045	0.902	**< 0.001**
		16	61	0.747^b^	0.611, 0.913	0.398	**< 0.001**	24	1.002	0.673, 1.492	0.144	0.067

Different superscript characters indicate significant differences among planting density under a given fertilization condition, and different subscript characters indicate significant differences among fertilization condition under a given planting density. Asterisks indicate cases where analysis was unavailable due to high plant mortality in a given treatment. Significant responses are highlighted in bold (*P* < 0.05).

### 3.4 Wheat and weeds allometric relationships between reproductive and vegetative biomass

The scaling exponents (α) for wheat vegetative biomass versus reproductive biomass were 0.965, 0.687, 0.677, 0.644 at planting densities of 4, 8, 12, 16, respectively, without fertilization. Under fertilization, the α values were 0.451 and 0.632 at planting densities of 12 and 16, respectively. For weeds, the α value was 1.138 at a planting density of 16 without fertilization. However, with fertilization, the α value was 0.337. At a planting density of 12 with fertilization, weeds had an α value of 0.920 (**Figure 3**, **Table 7**).

**Table 7:** Scaling exponents (α), their 95% confidence intervals (95% CI), and goodness of fit (R^2^) of wheat and weeds, based on standardized major axis (SMA) regression of log-transformed M_R_ and M_V_ under different fertilization condition (control and fertilization) and planting densities (4, 8, 12 and 16) at reproductive stage. N represents individual number. P values indicate the significance of SMA between the two organs.

Species	Treatment	Planting density	M_V_ vs M_R_ at reproductive stage				
			*N*	α	95% CI	*R^2^*	*P*
Wheat	Control	4	19	$0.965_{\text{A}}^{\text{a}}$	0.825, 1.128	0.906	**< 0.001**
		8	33	0.6877^b^	0.532, 0.886	0.504	**< 0.001**
		12	56	$0.677_{\text{A}}^{\text{b}}$	0.555, 0.826	0.463	**< 0.001**
		16	72	0.644^b^	0.553, 0.749	0.591	**< 0.001**
	Fertilization	4	18	$0.364_{\text{B}}^{\text{b}}$	0.223, 0.593	0.080	0.254
		8	32	-0.526^ab^	-0.756, -0.365	0.007	0.641
		12	53	$0.451_{\text{B}}^{\text{b}}$	0.350, 0.580	0.176	**< 0.01**
		16	60	0.632^a^	0.506, 0.789	0.277	**< 0.001**
Weeds	Control	4	2	*	*	*	*
		8	10	0.910	0.434, 1.909	0.013	0.757
		12	11	-1.309	-2.626, -0.652	0.000	0.949
		16	17	1.138_A_	0.715, 1.812	0.233	**< 0.05**
	Fertilization	4	7	0.623^ab^	0.236, 1.649	0.028	0.721
		8	11	0.493^ab^	0.265, 0.920	0.223	0.142
		12	17	0.920^a^	0.593, 1.427	0.322	**< 0.05**
		16	24	$0.337_{\text{B}}^{\text{b}}$	0.232, 0.489	0.259	**< 0.05**

Different superscript characters indicate significant differences among planting density under a given fertilization condition, and different subscript characters indicate significant differences among fertilization condition under a given planting density. Asterisks indicate cases where analysis was unavailable due to high plant mortality in a given treatment. Significant responses are highlighted in bold (*P* < 0.05).

## 4 Discussion

Our study found that aboveground, belowground, vegetative and reproductive biomass of wheat and weeds increased with fertilization, decreased with higher planting density, and exhibited limited interactive effects between these factors. For wheat biomass allocation, more biomass was allocated to roots than to shoots across ontogenetic stages and more to reproduction than vegetation at the reproductive stage. This allometric partitioning pattern remained consistent, regardless of fertilization or planting density, aligning with the allometric partitioning theory. In contrast, weeds biomass allocation strategies varied across fertilization and planting density gradients, indicating that weeds adjust biomass allocation in response to resource availability, consistent with the optimal partitioning theory.

Our findings align with previous studies on the effects of fertilization (Xia and Wan, 2008; Yue et al., 2016) and planting density (Fang et al., 1991) on plant biomass production. Fertilization enhanced aboveground, belowground, vegetative and reproductive biomass in both wheat and weeds, whereas higher planting density had negative effects, independent of ontogenetic stages. Increased N fertilizer application is expected to increase plant N uptake, enhance photosynthesis, and thereby improve biomass production (Lawlor et al., 2001). In contrast, increased plant density intensifies competition among individuals, modifying their structural and physiological characteristics by reducing resource availability per plant (Hecht et al., 2016; Postma et al., 2021; Rehling et al., 2021). Wheat exerted stronger competitive effects on weeds than weeds exerted on wheat, suggesting that wheat is a superior competitor, while weeds function as inferior competitors (**Tables 3**, **4**). This finding contributes to our understanding of species coexistence mechanisms in wheat-weed communities, particularly within the framework of contemporary species coexistence theory, which considers both intraspecific and interspecific competition (Chesson, 2000; Kraft et al., 2015; Van Dyke et al., 2022). Our research also revealed that biomass accumulation was driven by the additive effects of ontogenetic stages, fertilization, and planting density on biomass, rather than interactive effects, consistent with previous studies (Dieleman et al., 2012; Poorter et al., 2012). This suggests that each factor independently contributes to biomass accumulation without significant interactions. These additive effects imply that wheat production can be managed more flexibly in agricultural practice. By independently optimizing fertilization and planting density, wheat yield can be maximized, (Miguez et al., 2008).

**Figure 4:**
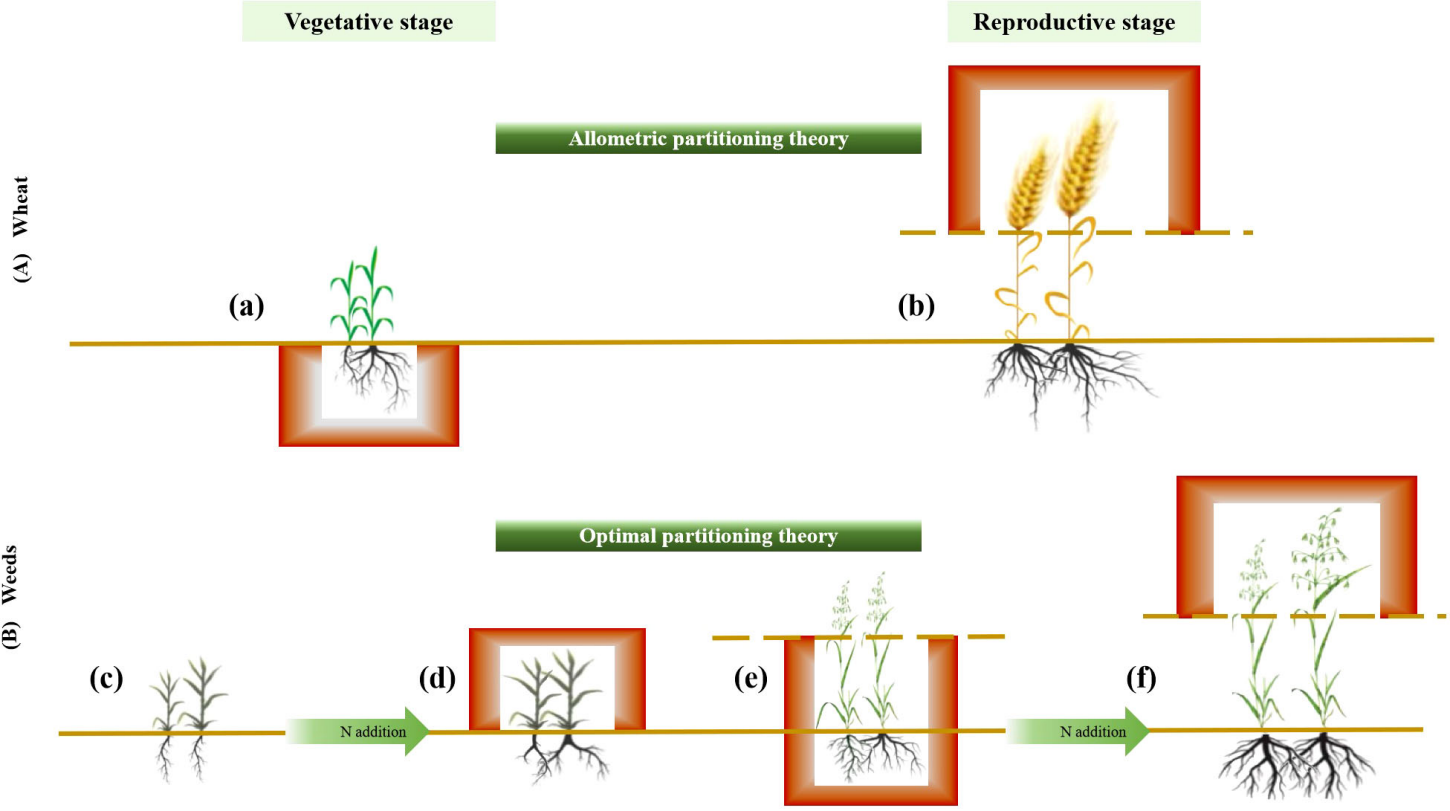
Diagram illustrating the effects of fertilization and planting density on the accumulation and allocation of aboveground, belowground, and reproductive biomass during the vegetative and reproductive stages in wheat and weeds. **(A)** Wheat follows the allometric partitioning theory, allocating more biomass to belowground than aboveground tissues during the vegetative stage (a) and more biomass to reproductive than vegetative structures during the reproductive stage (b), irrespective of fertilization and planting density. **(B)** Weeds exhibit allocation patterns consistent with the optimal partitioning theory, adjusting biomass distribution in response to resource availability. For example, at a density of 8 without fertilization, biomass was equally allocated between shoots and roots (c), whereas fertilization increased shoot allocation relative to root (d). At a density of 16 without fertilization, weeds allocated more biomass to vegetative growth than reproductive growth (e), but with fertilization, allocation shifted toward reproductive growth (f).

Standardized major axes regressions confirmed significant allometric scaling in wheat biomass distribution, indicating size-dependent allocation among aboveground, belowground, and reproductive components, consistent with the theory of allometric growth (**Figure 4**). Across gradients of fertilization and planting density, biomass allocation followed the same general allometric patterns: greater allocation to roots than shoots and greater allocation to reproduction than to vegetative structures. This suggests that while fertilization and planting density influence overall wheat biomass, the proportional allocation of biomass remains relatively fixed at a given plant size. Increased biomass allocation to roots suggests a greater limitation of water and nutrient than light, as root expansion is necessary for resource uptake, whereas light competition favors shoot growth (Padilla et al., 2009). During the reproductive phase, wheat prioritizes reproductive over vegetative biomass to ensure seed development and successful reproduction. This allocation strategy is likely a result of the “passive tendency” of this wheat variety to become a stable food crop after years of intensive human selection (Khan, 2016). Maintaining a balanced biomass distribution across roots, shoots, and reproductive organs is crucial for mechanical stability (Poorter et al., 2015). Future research on trait-size relationships in crop species will provide insights into how selective breeding for traits like high reproductive biomass can be harnessed to enhance crop yields (Westgeest et al., 2024).

Weeds demonstrate allocation strategies that align with the optimal partitioning theory, adjusting their allocation patterns in response to the environment to enhance survival and reproduction (**Figure 4**). This adaptability is consistent with their invasive nature in agricultural systems, where they thrive without structured planting schemes and remain highly dependent on environmental conditions (Ramesh et al., 2017). For example, at a density of 8 without fertilization, biomass allocation between shoots and roots was nearly equal. However, after fertilization, allocation shifted toward greater root biomass, likely due to increased nitrogen availability. Both light and nitrogen limitations promote a balanced shoot-to-root biomass allocation, whereas fertilization reduces belowground competition for nitrogen, allowing plants to focus on root expansion to exploit spatially heterogeneous soil resources (Cleland et al., 2019). At a density of 16, weeds allocated more biomass to vegetative growth under non-fertilized conditions, whereas fertilization shifted allocation toward seed production. This pattern reflects high phenotypic plasticity in growth and reproduction, enabling weeds to persist in nutrient-poor conditions (Garrison et al., 2024; Grime, 1977). As nutrient limitations are alleviated, weeds can allocate more resources to reproduction, allowing them to expand their population under favorable conditions while securing offspring production when resources are abundant.

Our findings have important implications for understanding species coexistence and phenotypic plasticity in wheat and weeds under abiotic stress. Conducting a reproductive fitness and whole life-history experiment provides a comprehensive, whole-plant perspective, which is essential for unravelling the physiological and biochemical mechanisms underlying adaptability. This approach also offers valuable insights for optimizing agricultural management practices to enhance crop resilience and productivity.

## Data Availability

The raw data supporting the conclusions of this article will be made available by the authors, without undue reservation.
