# Supplementary material for: Contrasting allocation patterns in wheat and weeds: allometric belowground and reproductive investment versus optimal partitioning adaptations
Source: Front Plant Sci. 2025 Apr 24;16:1542205. doi: 10.3389/fpls.2025.1542205 (PMC12060167; doi:10.3389/fpls.2025.1542205)
Supplement: Supplementary file 1 [file DataSheet1.docx]

Running head: Biomass allocation in wheat and weeds under fertilization and planting density.

Title: Contrasting allocation patterns in wheat and weeds: allometric belowground and reproductive investment versus optimal partitioning adaptations

**Jiazhen Xi^1^, Shengtao Shi^1^, Yizhong Rong^2^, Jie Liu^1^, Li Zhang^1*^**

**Table S1.** Experimental designed individual number (*n*) and actual individual number (*N*) of aboveground biomass (M_A_), belowground biomass (M_B_), reproductive biomass (M_R_) and vegetative biomass (M_V_) with different species (SP), fertilization (FT) and planting density (PD) at vegetative and reproductive stage. The difference between n and N comes from plant death

| **SP** | **FT** | **PD** | **Vegetative stage** | | **Reproductive stage** | | | |
| --- | --- | --- | --- | --- | --- | --- | --- | --- |
|  |  |  | **M_A_ and M_B_** | | **M_A_ and M_B_** | | **M_R_ and M_V_** | |
|  |  |  | ***n*** | ***N*** | ***n*** | ***N*** | ***n*** | ***N*** |
| Wheat | Control | 4 | 40 | 39 | 20 | 19 | 20 | 19 |
|  |  | 8 | 80 | 77 | 40 | 33 | 40 | 33 |
|  |  | 12 | 120 | 117 | 60 | 56 | 60 | 56 |
|  |  | 16 | 160 | 149 | 80 | 72 | 80 | 72 |
|  | N addition | 4 | 40 | 40 | 20 | 18 | 20 | 18 |
|  |  | 8 | 80 | 75 | 40 | 32 | 40 | 32 |
|  |  | 12 | 120 | 105 | 60 | 53 | 60 | 53 |
|  |  | 16 | 160 | 139 | 80 | 60 | 80 | 60 |
| Weeds | Control | 4 | 40 | 25 | 20 | 2 | 20 | 2 |
|  |  | 8 | 80 | 55 | 40 | 10 | 40 | 10 |
|  |  | 12 | 120 | 65 | 60 | 11 | 60 | 11 |
|  |  | 16 | 160 | 85 | 80 | 17 | 80 | 17 |
|  | N addition | 4 | 40 | 25 | 20 | 7 | 20 | 7 |
|  |  | 8 | 80 | 48 | 40 | 11 | 40 | 11 |
|  |  | 12 | 120 | 39 | 60 | 17 | 60 | 17 |
|  |  | 16 | 160 | 61 | 80 | 24 | 80 | 24 |

**Table S2.** Two-way ANOVA results of fertilization (FT), planting density (PD) and their interactions (A: Fertilization*Planting density) as well as one-way ANOVA results of planting density under different fertilization conditions (B: Planting density) on wheat's aboveground biomass (M_A_) and belowground biomass (M_B_) at vegetative and reproductive stage. Degrees-of-freedom (*df*), sum of squares (*Sum Sq*), mean square (*Mean Sq*), *F-values* and *P-values* are shown. Significant responses are highlighted in bold type (*P* < 0.05).

**A: Fertilization × Planting density**

| **Stage** | **Factors** | **M_A_** | | | | | **M_B_** | | | | |
| --- | --- | --- | --- | --- | --- | --- | --- | --- | --- | --- | --- |
|  |  | ***df*** | ***Sum Sq*** | ***Mean* *Sq*** | ***F*** | ***P*** | ***df*** | ***Sum Sq*** | ***Mean Sq*** | ***F*** | ***P*** |
| vegetative stage | FT | 1 | 5.605 | 5.605 | 84.321 | **< 0.001** | 1 | 3.027 | 3.027 | 19.928 | **< 0.001** |
|  | PD | 3 | 20.046 | 6.682 | 100.518 | **< 0.001** | 3 | 20.350 | 6.783 | 44.661 | **< 0.001** |
|  | FT: PD | 3 | 0.107 | 0.036 | 0.538 | 0.656 | 3 | 0.133 | 0.044 | 0.293 | 0.831 |
|  | Residuals | 733 | 48.727 | 0.067 |  |  | 733 | 111.330 | 0.152 |  |  |
| reproductive stage | FT | 1 | 1.861 | 1.861 | 34.354 | **< 0.001** | 1 | 4.369 | 4.369 | 39.543 | **< 0.001** |
|  | PD | 3 | 8.015 | 2.672 | 49.330 | **< 0.001** | 3 | 11.979 | 3.993 | 36.140 | **< 0.001** |
|  | FT: PD | 3 | 0.067 | 0.022 | 0.413 | 0.744 | 3 | 0.455 | 0.152 | 1.372 | 0.251 |
|  | Residuals | 335 | 18.143 | 0.054 |  |  | 335 | 37.013 | 0.111 |  |  |

**B: Planting density**

| **Stage** | **Factors** | | **M_A_** | | | | | **M_B_** | | | | |
| --- | --- | --- | --- | --- | --- | --- | --- | --- | --- | --- | --- | --- |
|  |  |  | ***df*** | ***Sum Sq*** | ***Mean Sq*** | ***F*** | ***P*** | ***df*** | ***Sum Sq*** | ***Mean Sq*** | ***F*** | ***P*** |
| vegetative stage | Control | PD | 3 | 10.219 | 3.406 | 57.202 | **< 0.001** | 3 | 10.284 | 3.428 | 32.061 | **< 0.001** |
|  |  | Residuals | 378 | 22.509 | 0.060 |  |  | 378 | 40.416 | 0.107 |  |  |
|  | N addition | PD | 3 | 9.935 | 3.312 | 44.840 | **< 0.001** | 3 | 10.199 | 3.400 | 17.019 | **< 0.001** |
|  |  | Residuals | 355 | 26.218 | 0.074 |  |  | 355 | 70.914 | 0.200 |  |  |
| reproductive stage | Control | PD | 3 | 3.568 | 1.190 | 22.189 | **< 0.001** | 3 | 5.662 | 1.887 | 17.761 | **< 0.001** |
|  |  | Residuals | 176 | 9.434 | 0.054 |  |  | 176 | 18.702 | 0.106 |  |  |
|  | N addition | PD | 3 | 4.514 | 1.505 | 27.469 | **< 0.001** | 3 | 6.772 | 2.257 | 19.601 | **< 0.001** |
|  |  | Residuals | 159 | 8.709 | 0.055 |  |  | 159 | 18.311 | 0.115 |  |  |

**Table S3.** Two-way ANOVA results of fertilization (FT), planting density (PD) and their interactions (A: Fertilization*Planting density) as well as one-way ANOVA results of planting density under different fertilization conditions (B: Planting density) on weeds' aboveground biomass (M_A_) and belowground biomass (M_B_) at vegetative and reproductive stage. Degrees-of-freedom (*df*), sum of squares (*Sum Sq*), mean square (*Mean Sq*), *F-values* and *P-values* are shown. Significant responses are highlighted in bold (*P* < 0.05).

**A: Fertilization × Planting density**

| **Stage** | **Factors** | **M_A_** | | | | | **M_B_** | | | | |
| --- | --- | --- | --- | --- | --- | --- | --- | --- | --- | --- | --- |
|  |  | ***df*** | ***Sum Sq*** | ***Mean Sq*** | ***F*** | ***P*** | ***df*** | ***Sum Sq*** | ***Mean Sq*** | ***F*** | ***P*** |
| vegetative stage | FT | 1 | 10.305 | 10.305 | 23.217 | **< 0.001** | 1 | 3.956 | 3.956 | 10.673 | **< 0.001** |
|  | PD | 3 | 10.350 | 3.450 | 7.773 | **< 0.001** | 3 | 4.313 | 1.438 | 3.879 | **< 0.001** |
|  | FT: PD | 3 | 0.295 | 0.098 | 0.222 | 0.881 | 3 | 0.312 | 0.104 | 0.280 | 0.840 |
|  | Residuals | 395 | 175.320 | 0.444 |  |  | 395 | 146.391 | 0.371 |  |  |
| reproductive stage | FT | 1 | 0.449 | 0.449 | 2.786 | 0.099 | 1 | 0.001 | 0.001 | 0.005 | 0.945 |
|  | PD | 3 | 6.171 | 2.057 | 12.769 | **< 0.001** | 3 | 4.541 | 1.514 | 10.206 | **< 0.001** |
|  | FT: PD | 3 | 0.126 | 0.042 | 0.261 | 0.853 | 3 | 0.471 | 0.157 | 1.058 | 0.371 |
|  | Residuals | 91 | 14.659 | 0.161 |  |  | 91 | 13.497 | 0.148 |  |  |

**B: Planting density**

| **Stage** | **Factors** | | **M_A_** | | | | | **M_B_** | | | | |
| --- | --- | --- | --- | --- | --- | --- | --- | --- | --- | --- | --- | --- |
|  |  |  | ***df*** | ***Sum Sq*** | ***Mean Sq*** | ***F*** | ***P*** | ***df*** | ***Sum Sq*** | ***Mean Sq*** | ***F*** | ***P*** |
| vegetative stage | Control | PD | 3 | 4.563 | 1.521 | 3.002 | **< 0.05** | 3 | 2.485 | 0.828 | 1.779 | 0.152 |
|  |  | Residuals | 226 | 114.527 | 0.507 |  |  | 226 | 105.214 | 0.466 |  |  |
|  | N addition | PD | 3 | 6.081 | 2.027 | 5.635 | **< 0.01** | 3 | 2.139 | 0.713 | 2.927 | **< 0.05** |
|  |  | Residuals | 169 | 60.792 | 0.360 |  |  | 169 | 41.177 | 0.244 |  |  |
| reproductive stage | Control | PD | 3 | 1.927 | 0.642 | 3.548 | **< 0.05** | 3 | 1.676 | 0.559 | 3.506 | **< 0.05** |
|  |  | Residuals | 36 | 6.518 | 0.181 |  |  | 36 | 5.738 | 0.159 |  |  |
|  | N addition | PD | 3 | 4.370 | 1.457 | 9.840 | **< 0.001** | 3 | 3.335 | 1.112 | 7.881 | **< 0.001** |
|  |  | Residuals | 55 | 8.141 | 0.148 |  |  | 55 | 7.759 | 0.141 |  |  |

**Table S4.** Two-way ANOVA results of fertilization (FT), planting density (PD) and their interactions (A: Fertilization*Planting density) as well as one-way ANOVA results of planting density under different fertilization conditions (B: Planting density) on wheat and weeds' reproductive biomass (M_R_) and vegetative biomass (M_V_) at reproductive stage. Degrees-of-freedom (*df*), sum of squares (*Sum Sq*), mean square (*Mean Sq*), *F-values* and *P-values* are shown. Significant responses are highlighted in bold(*P* < 0.05).

**A: Fertilization × Planting density**

| **Species** | **Factors** | **M_R_** | | | | | **M_V_** | | | | |
| --- | --- | --- | --- | --- | --- | --- | --- | --- | --- | --- | --- |
|  |  | ***df*** | ***Sum Sq*** | ***Mean Sq*** | ***F*** | ***P*** | ***df*** | ***Sum Sq*** | ***Mean Sq*** | ***F*** | ***P*** |
| wheat | FT | 1 | 0.007 | 0.007 | 0.049 | 0.826 | 1 | 3.317 | 3.317 | 67.317 | **< 0.001** |
|  | PD | 3 | 7.357 | 2.452 | 16.821 | **< 0.001** | 3 | 8.319 | 2.773 | 56.277 | **< 0.001** |
|  | FT: PD | 3 | 0.451 | 0.151 | 1.032 | 0.378 | 3 | 0.265 | 0.088 | 1.792 | 0.148 |
|  | Residuals | 335 | 48.842 | 0.146 |  |  | 335 | 16.508 | 0.049 |  |  |
| weeds | FT | 1 | 0.812 | 0.812 | 2.349 | 0.129 | 1 | 0.229 | 0.229 | 1.498 | 0.224 |
|  | PD | 3 | 9.132 | 3.044 | 8.800 | **< 0.001** | 3 | 5.399 | 1.800 | 11.785 | **< 0.001** |
|  | FT: PD | 3 | 0.469 | 0.156 | 0.452 | 0.716 | 3 | 0.211 | 0.070 | 0.462 | 0.710 |
|  | Residuals | 91 | 31.476 | 0.346 |  |  | 91 | 13.896 | 0.153 |  |  |

**B: Planting density**

| **Species** | **Factors** | | **M_R_** | | | | | **M_V_** | | | | |
| --- | --- | --- | --- | --- | --- | --- | --- | --- | --- | --- | --- | --- |
|  |  |  | ***df*** | ***Sum Sq*** | ***Mean Sq*** | ***F*** | ***P*** | ***df*** | ***Sum Sq*** | ***Mean Sq*** | ***F*** | ***P*** |
| wheat | Control | PD | 3 | 5.680 | 1.893 | 19.746 | **< 0.001** | 3 | 3.042 | 1.014 | 22.392 | **< 0.001** |
|  |  | Residuals | 176 | 16.874 | 0.096 |  |  | 176 | 7.970 | 0.045 |  |  |
|  | N addition | PD | 3 | 2.129 | 0.710 | 3.530 | **< 0.05** | 3 | 5.542 | 1.847 | 34.407 | **< 0.001** |
|  |  | Residuals | 159 | 31.968 | 0.201 |  |  | 159 | 8.537 | 0.054 |  |  |
| weeds | Control | PD | 3 | 2.520 | 0.840 | 5.232 | **< 0.01** | 3 | 1.878 | 0.626 | 3.199 | **< 0.05** |
|  |  | Residuals | 36 | 5.779 | 0.161 |  |  | 36 | 7.044 | 0.196 |  |  |
|  | N addition | PD | 3 | 7.081 | 2.360 | 5.052 | **< 0.01** | 3 | 3.733 | 1.244 | 9.988 | **< 0.001** |
|  |  | Residuals | 55 | 25.698 | 0.467 |  |  | 55 | 6.852 | 0.125 |  |  |


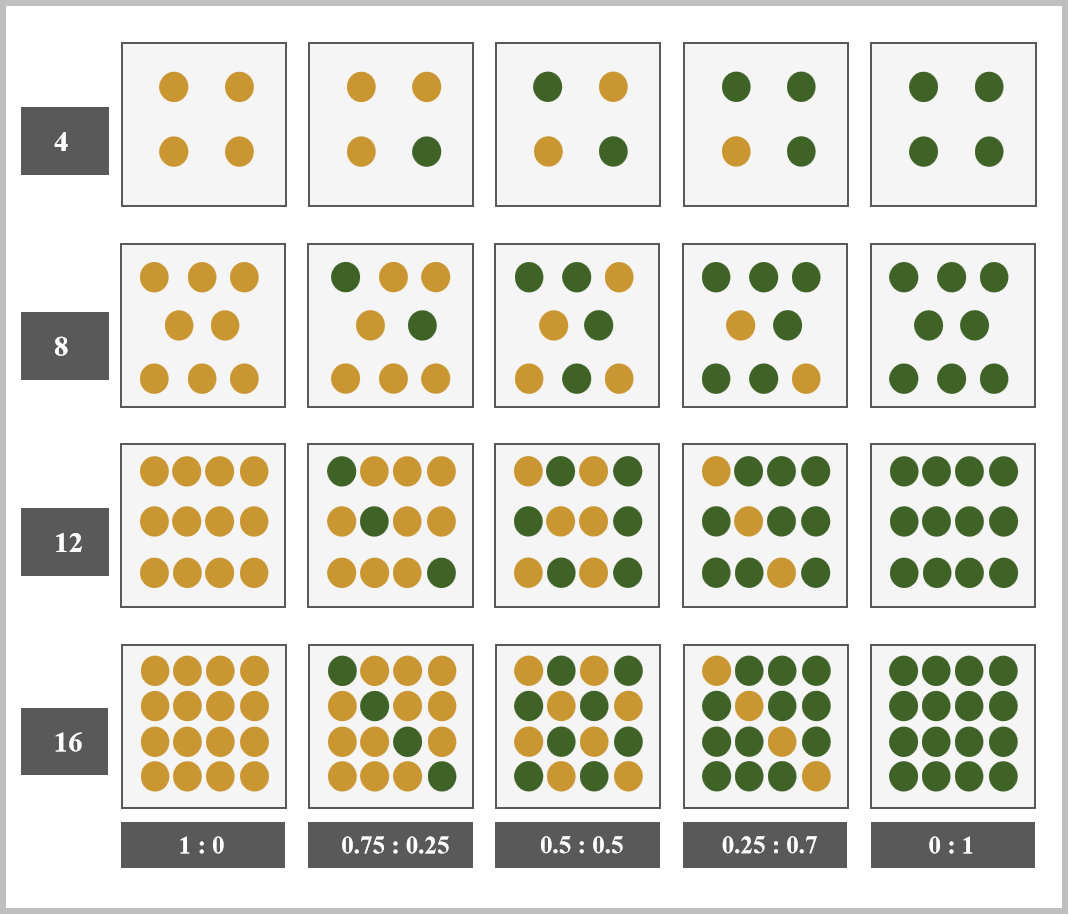


**Figure S1. Planting design of one competing pair.** The experiment included six groups; half received urea while the other half were controls. Each group consisted of 40 pots and explored the interaction between two competing plant pairs: *T. aestivum* and *A. fatua*, as well as *T. aestivum* and *E. crusgalli*. For each competing pair, the yellow dots represent wheat, and green dots represent weeds with planting densities of 4, 8, 12, or 16 individuals per pot. The wheat-to-weed ratios were 1: 0, 0.75:0.25, 0.5:0.5, 0.25:0.75, and 0:1, simulating the species diversity found in natural wheat field communities. For both urea addition and control groups, biomass was measured at both vegetative (2 groups) and reproductive stage (1 group). That is, (20 pots *2 competing pairs) * Urea addition (presence and absence) * (2 vegetative stage+ 1 reproductive stage).
